# Development of a Portable Motor Learning Laboratory (PoMLab)

**DOI:** 10.1371/journal.pone.0157588

**Published:** 2016-06-27

**Authors:** Ken Takiyama, Masahiro Shinya

**Affiliations:** 1 Department of Electrical and Electronic Engineering, Tokyo University of Agriculture and Technology, Tokyo, Japan; 2 Department of Life Sciences, Graduate School of Arts and Sciences, The University of Tokyo, Tokyo, Japan; VU University Amsterdam, NETHERLANDS

## Abstract

Most motor learning experiments have been conducted in a laboratory setting. In this type of setting, a huge and expensive manipulandum is frequently used, requiring a large budget and wide open space. Subjects also need to travel to the laboratory, which is a burden for them. This burden is particularly severe for patients with neurological disorders. Here, we describe the development of a novel application based on Unity3D and smart devices, e.g., smartphones or tablet devices, that can be used to conduct motor learning experiments at any time and in any place, without requiring a large budget and wide open space and without the burden of travel on subjects. We refer to our application as POrtable Motor learning LABoratory, or PoMLab. PoMLab is a multiplatform application that is available and sharable for free. We investigated whether PoMLab could be an alternative to the laboratory setting using a visuomotor rotation paradigm that causes sensory prediction error, enabling the investigation of how subjects minimize the error. In the first experiment, subjects could adapt to a constant visuomotor rotation that was abruptly applied at a specific trial. The learning curve for the first experiment could be modeled well using a state space model, a mathematical model that describes the motor leaning process. In the second experiment, subjects could adapt to a visuomotor rotation that gradually increased each trial. The subjects adapted to the gradually increasing visuomotor rotation without being aware of the visuomotor rotation. These experimental results have been reported for conventional experiments conducted in a laboratory setting, and our PoMLab application could reproduce these results. PoMLab can thus be considered an alternative to the laboratory setting. We also conducted follow-up experiments in university physical education classes. A state space model that was fit to the data obtained in the laboratory experiments could predict the learning curves obtained in the follow-up experiments. Further, we investigated the influence of vibration function, weight, and screen size on learning curves. Finally, we compared the learning curves obtained in the PoMLab experiments to those obtained in the conventional reaching experiments. The results of the in-class experiments show that PoMLab can be used to conduct motor learning experiments at any time and place.

## Introduction

When we are challenged with a new sport or rehabilitation, the desired performance cannot be achieved at first. As we practice, we can gradually improve our motor skills, and eventually we can achieve the desired performance. This learning process, in which motor skill gradually improves, is called motor learning. Because motor learning is believed to originate in plasticity in the brain [1,2], many researchers over many years have conducted motor learning experiments using patients (e.g., stroke patients) [3], the elderly [4], children [5], or healthy subjects [6,7] to understand the neural mechanisms of motor learning.

Paradigms that involve reaching movements and perturbation learning, such as curl force field [6] or visuomotor rotation [7,8], are a popular way of investigating motor learning. In such experiments, subjects are seated in a chair in front of a monitor while holding a manipulandum. The manipulandum operates similarly to a computer mouse: if the subject moves the manipulandum in a forward direction, the cursor displayed on the monitor moves accordingly. By controlling the manipulandum, the subjects are instructed to move the cursor towards a displayed target in as direct a path as possible within an appropriate length of time, e.g., within 500±50 msec [9]. By employing these fast reaching movements, conventional experiments have investigated motor learning by measuring how subjects updated motor commands to decrease the prediction error, a discrepancy between the predicted and actual cursor movement. The prediction error is introduced during a specific trial without any notification via the application of a perturbation. For example, in a clockwise (CW) visuomotor rotation condition, when a subject manipulates the manipulandum to cause the cursor to move accordingly, the cursor movement deflects from the assumed movement in a CW direction. In the absence of motor command updating, subjects cannot move the cursor towards the target and cannot successfully accomplish the imposed task. Paradigms that involve reaching movements and perturbation learning enable the investigation of how subjects update motor commands, adapt to the perturbation, and decrease the prediction error [9]. The adaptation process includes at least two processes, minimization of the prediction error and forgetting of motor memory [10]; here, we refer to these processes as the motor learning process.

This type of paradigm also enables comparison of the motor learning process of patients with that of healthy subjects, which facilitates understanding of how particular brain regions are related to the motor learning process. For example, stroke patients show slower learning speeds than control subjects [3], patients with Parkinson’s disease show worse consolidation than control subjects [5], cerebellar ataxia patients can better adapt to small perturbations than to large ones [11], and patients with Huntington’s disease have an intact ability to adapt to such perturbations [12]. In addition, to investigate the relationship between development and the motor learning process, motor learning experiments have also been conducted using the elderly and children as subjects. Elderly individuals whose declarative memory capacity is good show motor learning abilities that are similar to those of young adults, but elderly individuals who have memory capacity deficits show poor motor learning abilities compared to young adults [4]. Additionally, motor learning experiments are utilized to identify the relationships between social communication abilities and motor learning, e.g., children with autism and children without autism show comparable motor learning abilities [5]. Notably, these important findings were reported based on laboratory experiments. Laboratory experiments with manipulandums enable the data to be precisely and reliably measured.

The manipulandums used to conduct this type of experiment are large and expensive. As a result, the experiments require a large budget and wide open space; therefore, the experiments often can be conducted only in limited locations, i.e., laboratories. Further, laboratory experiments require the patients, elderly, and children who serve as subjects to travel to the laboratory, which is a large burden for them, especially for patients. Thus, it is difficult to conduct laboratory experiments using a sufficient number of patients. Because each patient exhibits different symptoms, it is desirable to investigate individual differences in the motor learning process among patients. To investigate individual differences, the motor learning experiments should be conducted using a large number of patients. In addition, some motor learning experiments are designed to investigate individual differences in healthy subjects [13]. Even in healthy subjects, it is necessary to employ many subjects in the experiments to investigate inter-individual differences, and this requires a considerable amount of time because each subject must travel to the laboratory and because the experiments must be conducted one subject at a time in a laboratory setting. The constraints of laboratory experiments, including the money, space, and budget required as well as the burden for the subjects to travel to the laboratory, prevent experimenters from conducting motor learning experiments using a sufficient number of subjects.

Here, we develop a novel tool to conduct motor learning experiments anytime and anywhere through the use of smart devices (e.g., smartphones and tablets). There is no doubt that in recent years, a huge number of people use smartphones and tablets at all times and places. Thus, we developed an application to conduct motor learning experiments that works on smart devices. Our application enables one to conduct motor learning experiments without the need for any expensive devices beyond the smart device. The experiments are also not limited to a laboratory; instead, they can be conducted anywhere, such as a home or hospital, and at any time that is suitable for the subjects. Especially for patients, these advantages can decrease the burden of traveling to the laboratory. We developed the application using the game developing tool Unity3D. Unity3D enables one to develop applications that are independent of a specific operating system or device, i.e., we could develop a multiplatform application. Unity3D enables the application to be freely shared not only with researchers in the field of motor learning but also with non-researchers, doctors, and therapists. The convenience and portability of the application as well as its ability to be shared can be expected to enable the investigation of the motor leaning process in many healthy subjects, patients, elderly individuals, and children, anywhere and at any time. In fact, in recent years, Unity3D has been used to develop new applications for stroke rehabilitation [14] and sports training [15]. In short, by utilizing smart devices and Unity3D, we developed a multiplatform application that enables the conduction of motor learning experiments anywhere and at any time, decreasing the burden for patients. The application is also available and sharable for free. Subsequently, we refer to our application as POrtable Motor learning LABoratory, or PoMLab.

First, we provide a detailed description of PoMLab in the *Materials & methods* section. Second, in the *Results* section, we describe the results of PoMLab experiments that we conducted to determine whether our PoMLab application could be an alternative to conventional laboratory experiments of motor learning. More specifically, we conducted a behavioral experiment in which subjects were required to adapt to a constant visuomotor rotation that was applied during a specific trial without any notification. Because state space models are widely used to explain learning curves obtained through experiments with reaching movements [9,16], we evaluated whether a state space model could be fit to the learning curve obtained in the PoMLab experiment. Further, we determined whether the state space model could predict the learning curves obtained in a second experiment. In the second experiment, subjects experienced a perturbation that gradually increased each trial. Because a previous experiment that utilized reaching movements reported that subjects can adapt to the gradually increasing perturbation without being aware of the perturbation [17], we also evaluated whether the subjects in the PoMLab experiment could adapt to the perturbation without any awareness. Third, to confirm whether the PoMLab enables data to be obtained at any time and place, we conducted follow-up experiments (60 subjects for 7 hours). We determined whether a state space model that was fit to data obtained from a laboratory experiment could predict the learning curves obtained in the follow-up experiments. Because several kinds of smart devices with different properties are available, we further investigated the influence of the vibration function, weight, and screen size of tablet devices on the learning curves. Finally, we compared the learning curves obtained in the PoMLab experiments to those obtained in the conventional reaching experiments.

## Materials & Methods

### Smart device & computer settings

The PoMLab application was developed using an Android tablet (Nexus 9, HTC, Taipei City, Taiwan, 2048×1536 pixels, 228.25×153.68×7.95 mm screen size, and 436 g weight). Using a personal edition of Unity (version 5.2), one author used a Mac personal computer (OS X 10.10, OS X Yosemite), and the other author used a Windows personal computer (Windows 7, 64bit) to develop PoMLab. In the laboratory experiments, Nexus 9 tablets were used. In the follow-up experiments, Nexus 9 tablets were used for 48 subjects and Memo Pad 7 tablets (ASUSTeK Computer Inc., Taipei, China, 1280×800 pixels, 189.3×113.7×9.6 mm screen size, and 295 g weight) were used for 12 subjects. We confirmed that the PoMLab worked on another Android tablet (Kindle Fire HDX, Amazon.com, Seattle, United States).

#### Participants in the laboratory experiments

Twenty-two healthy volunteers (20 males, 2 females, aged 22–28 years) participated in the laboratory experiments in this study (12 in the first experiment, and 10 in the second experiment). Each subject came to our laboratory. The subjects were pseudo-randomly assigned to one of the two experimental groups: the CW visuomotor rotation group or the counter-clockwise (CCW) visuomotor rotation group. In the CW group, cursor movements in trials with visuomotor rotation were deflected from the path that corresponded to the same movements in control trials in a clockwise direction. The movement error *e* and the perturbation *r* were defined to be positive in the CW rotation condition and negative in the CCW condition. Because the visuomotor rotation could be defined as either CW or CCW, we calculated the average learning performance across the two visuomotor rotation conditions. The subjects had no cognitive or motor disorders and were naive to the concept of visuomotor rotation and the purpose of the experiment. All procedures were approved by the ethics committee of Tokyo University of Agriculture and Technology. The subjects were paid for their time.

All subjects throughout all experiments were clearly informed of the experimental procedures in accordance with the Declaration of Helsinki and provided written informed consent before the experiment began.

#### Participants in the follow-up experiments

Sixty healthy undergraduate students (44 males, 16 females, aged 18–23 years) participated in the follow-up experiments in this study. The follow-up experiments were conducted in three classrooms. In each room, a teaching assistant (graduate school student) ran the experiment, including providing instructions about the operation of the device. When two participants performed the task in a room at the same time, they sat back-to-back so that they were not bothered by the other participant. The subjects were pseudo-randomly assigned to either the CW or the CCW visuomotor rotation group. The subjects had no cognitive or motor disorders and were naive to the concept of visuomotor rotation and the purpose of the experiment. All procedures were approved by the ethics committee of the University of Tokyo.

### Setting of the PoMLab experiments

**Fig 1:**
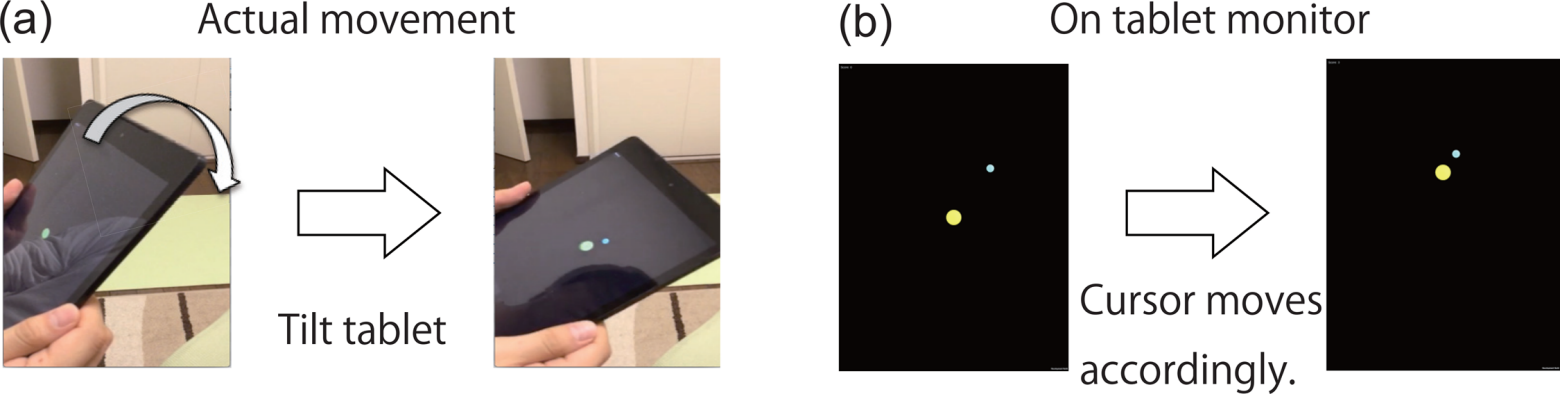
Summary of the PoMLab experiment. (a): Actual movements that the subjects were required to do in the PoMLab experiments. The subjects were required to tilt the tablet device to move the cursor on the screen. (b): Cursor movements on the tablet monitor. When the subjects tilted the tablet forward, the cursor on the tablet monitor moved in an upward direction. This relationship between the direction of tilting and the direction of resulting cursor movement changed in trials with visuomotor rotation.

**Fig 2:**
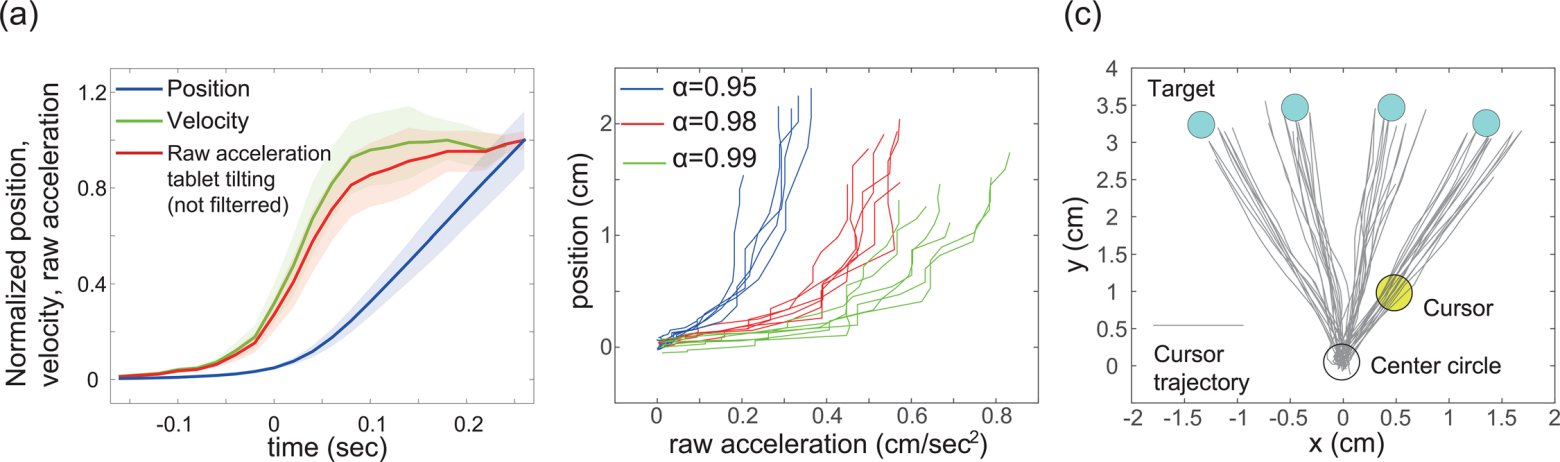
Kinematics of a typical subject in a PoMLab experiment with a Nexus 9. (a): Typical kinematics in the PoMLab experiments. This figure shows the kinematics for a target direction of 82.5°. The blue and green lines depict the temporal evolution of the cursor position and velocity, respectively (mean±s.e.m. calculated for 8 trials). The red line depicts the acceleration of tablet tilting. Those values were normalized by their own maximum values. We used the Unity function "Input.acceleration" in the FixedUpdate function with a FixedDeltaTime of 0.02 seconds to detect the acceleration, and the cursor position was determined based on the acceleration value. Because we used a low-pass filter when determining the relationship between tilting acceleration and cursor movement, the relationship between these values includes a time lag. To draw this figure, we detected the onset of movement (time = 0 on the x-axis) as the time at which the distance between the center circle and the cursor first exceeded 0.1 cm. This figure shows each kinematic value from -0.15 seconds to 0.26 seconds. (b): Influence of the low-pass filter parameter on the relationship between the raw acceleration value and the displayed cursor position. This figure shows the kinematics in eight trials for a target direction of 82.5°. The eight blue, red, and green lines depict the relationship when α = 0.95, 0.98, and 0.99 in each trial. (c): Trajectories of the center of the cursor. Each gray line depicts the cursor trajectory during a single trial. The target amplitude was 3.5 cm for the Nexus 9; this value remained constant across all of the target directions. The target directions were sampled from among 67.5°, 82.5°, 97.5°, and 112.5° in a pseudo-random manner, i.e., each target direction was sampled once every four trials. The white, yellow, and blue circles denote the center circle, the cursor, and the target, respectively.

Subjects sat in a chair and held an Android tablet using both hands. In PoMLab, a cursor (yellow circle) was displayed on the tablet monitor, and the subjects were required to control the cursor by tilting the tablet (Fig 1). The subjects needed to move the cursor by tilting the tablet forward, backward, rightward and leftward, i.e., PoMLab is a two-dimensional task. We used the tilting task because it can be completed anywhere. For example, pointing movements on a touchscreen require a desk on which to place the tablet. Further, in contrast to conventional motor learning experiments, visuomotor rotation is easily recognized in pointing tasks because the subjects can judge the difference between their visible fingers and the displayed cursor. Translating a tablet is another potential candidate method, but a sufficiently large space is required. In contrast, the tilting task can be conducted in a small workspace, and visuomotor rotation can be introduced without the subject’s awareness. We used the command "Input.acceleration" in the FixedUpdate function in Unity3D, which detected the linear acceleration of the tilting every 20 ms (50 Hz). The subjects always moved the tablet slightly, and these slight movements caused oscillatory movements of the cursor. To eliminate the oscillatory movements, we used a simple low-pass filter to transform the raw acceleration information at time *t*, *a*(*t*) = (*a*_*x*_(*t*), *a*_*y*_(*t*)), into the cursor position at time *t*, *p*(*t*) = (*p*_*x*_(*t*), *p*_*y*_(*t*)), according to the equation *p*(*t*) = α*p*(*t*)+(1−α)*a*(*t*), where α is set to 0.98 (an example of the relationship between the acceleration and the cursor position is shown in Fig 2A). The cursor position data *p(t)* were updated using the Update method in Unity3D. Fig 2B shows the influence of the parameter α on the relationship between raw acceleration and cursor position. A larger α resulted in a slower influence of acceleration on the cursor position.

At the beginning of each trial, a center circle (white circle) was displayed (Fig 2C). After the yellow cursor remained on the center circle for more than two seconds, a target (cyan circle) appeared. This experimental setting enabled the initial position of the yellow cursor to be fixed, which enabled the determination of how the cursor position deviated from a straight line between the center circle and the position of the target. In our experimental setting, the target amplitude was set to four in Unity 3D (corresponding to a distance between the center circle and the targets of 3.5 cm and 3.0 cm on the Nexus 9 and Memo Pad 7, respectively), and target angles were pseudo-randomly sampled from 67.5°, 82.5°, 97.5°, or 112.5°, i.e., each target angle was sampled once every four trials. We used these four upwardly positioned targets because tilting forward is easier than tilting leftward or rightward, likely due to the inertia of the tablet and our arms. Further, tilting backward is more likely to cause the tablet to slip from our hands than tilting forward. Although eight radially distributed targets are widely used in conventional experiments, we used the four upwardly positioned targets. These target angle settings enabled exclusion of the possibility that the subjects could predict the position of the target in the next trial. A group of four trials, each with a different target angle, is subsequently referred to as one set. Fig 2C shows the size of the center circle, the cursor, the target, the detected cursor movements, and each target’s position on the screen of a Nexus 9.

The subjects were asked to move the yellow cursor in as straight a path as possible towards the target within 1.5 seconds. After 1.5 seconds, the target automatically disappeared, and the center circle re-appeared. When the cursor reached the target, the target disappeared, a small animation of an explosion appeared at the target’s previous location. The tablet also vibrated if it had the capacity to do so; the Nexus 9 includes a vibratory function, but the Memo Pad 7 does not. We compared the learning curves of subjects who used tablets with and without vibration in the *Influence of vibration* section. Contact between the cursor and the target was detected using the Unity function "collider" (we used sphere collider for the cursor, target, and center circle). According to our settings, contact was detected when a portion of the cursor overlapped with a portion of the cursor; as a result, contact was detected even when there was 7.38° and 7.59° of deviation from a straight line between the center circle and the target on the Nexus 9 and Memo Pad 7, respectively.

### Setting of the manipulandum experiments

#### Participants and experimental setting

Fourteen healthy, right-handed volunteers (9 males and 5 females, aged 19–31 years) participated in the PoMLab experiments and the experiments with the manipulandum (Phantom 1.5 HF; Geomagic, Rock Hill, SC, USA). Seven subjects underwent the PoMLab experiments followed by experiments with the manipulandum. The other subjects underwent experiments with the manipulandum first followed by the PoMLab experiments. The subjects were asked to make pointing movements with their right arm while holding the handle of the manipulandum. The handle position was displayed as a yellow cursor (a 10-mm circle) on a black background on a horizontal screen located above their hand. The movement of the handle was constrained to a virtual horizontal plane (10 cm below the screen) that was implemented by a simulated spring (1.0 kN/m) and dumper (0.1 N/[m/s]). A brace was used to reduce unwanted wrist movement. Upper trunk motion was constrained by a harness. Before each trial, the participants were required to hold the cursor at its starting position (an 8-mm white circle). After a 2-s holding time, a cyan target (an 8-mm circle) appeared. Immediately after the target appeared, the participant needed to initiate a pointing movement. The subjects were required to move the handle with a peak velocity of 376±36 mm/s (the target velocity was calculated according to minimum-jerk theory with a movement amplitude of 14 cm and a duration of 700 msec). A warning message appeared on the screen if the movement velocity of the handle rose above ("Fast") or fell below ("Slow") this threshold value. The subjects were also required to move the handle with an amplitude of 7 cm and return it to the center position at once, in the so-called out-back reaching movement. When the yellow cursor touched the target, the target disappeared. These settings were similar to those used in the PoMLab experiments. Seven subjects experienced the experiment with reaching movements first and then experienced the PoMLab experiment. Three of these subjects experienced CW rotation in both experiments, while the other subjects experienced CCW rotation. The other seven subjects experienced the PoMLab experiment first and then the experiment with reaching movements. Three of these subjects experienced CW rotation in both experiments, and the other subjects experienced CCW rotation.

#### Data recordings

The manipulandum motion data were recorded at a sampling rate of 500 Hz and low-pass filtered using a fourth-order Butterworth filter with a 10-Hz cutoff. Movement onset time was defined as the first time point in the first time period during which hand movement velocity first exceeded 10% of its peak value for at least 50 ms.

## Experimental Results

First, to validate that the PoMLab application could be an alternative to conventional motor learning experiments, we determined whether using PoMLab to conduct experiments in the laboratory could reproduce experimental results obtained for reaching movements and perturbation learning. Second, to confirm that PoMLab could be used to measure motor learning data at any time or place, we conducted follow-up experiments and compared the data to those obtained in the laboratory setting. Finally, we compared the learning curves obtained in the PoMLab experiments to those obtained in conventional reaching experiments.

### Laboratory experiment

#### Adaptation to constant perturbation

In the first experiment, subjects completed 32 practice trials, or 8 practice sets. After the practice trials, the subjects completed 12 baseline trials, followed by 40 trials with constant visuomotor rotation of 15° or -15°. The visuomotor rotation was abruptly applied at the 13th trial, which corresponded to the beginning of the 4th set, without any notification. Half of the subjects (6 subjects) experienced a CW visuomotor rotation of 15°, and the other subjects (6 subjects) experienced a CCW visuomotor rotation of -15°.

**Fig 3:**
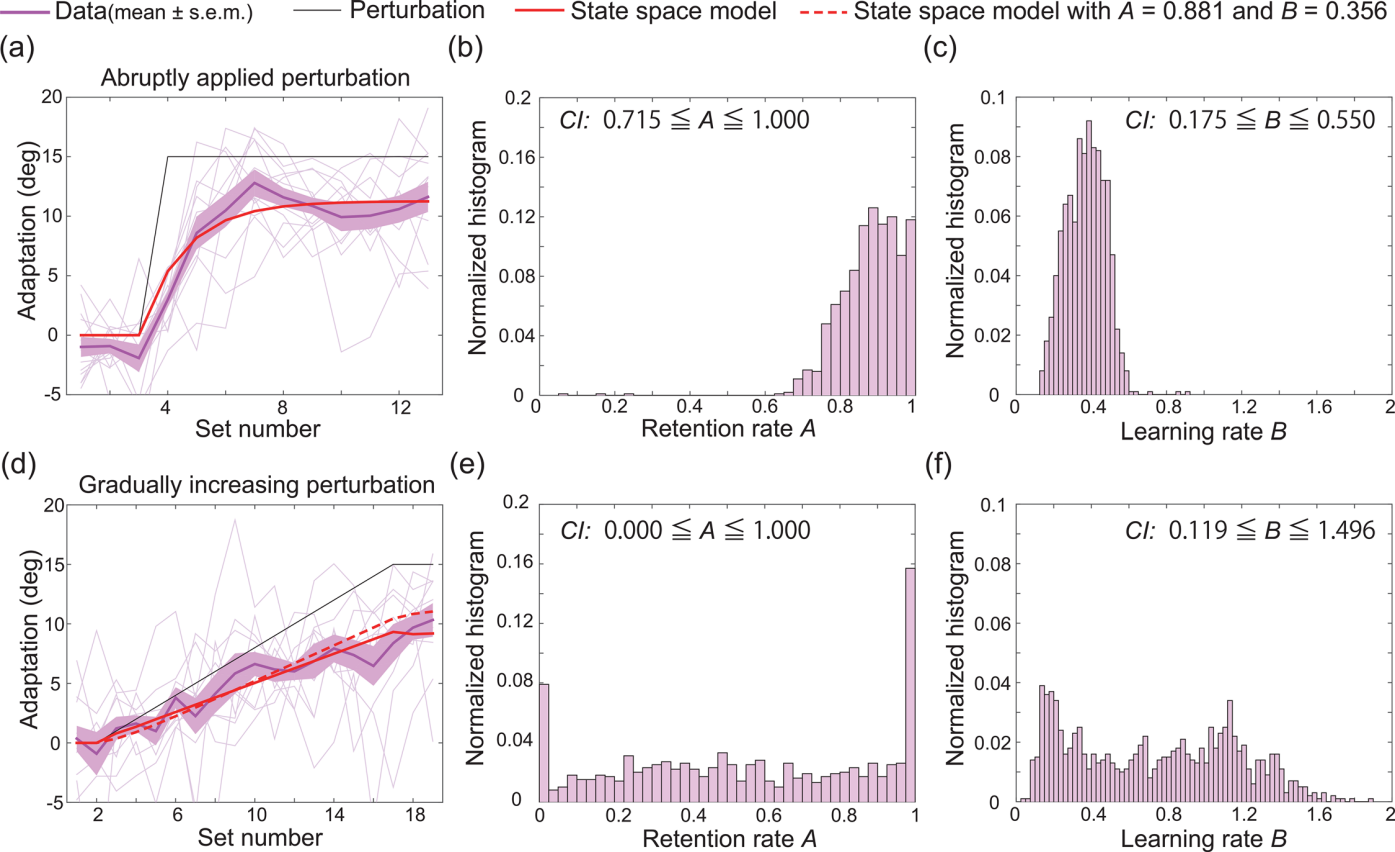
Results of the first and second laboratory experiments. (a): Results of the first experiment. The horizontal and vertical axes denote the set number and the degree of adaptation, respectively (see *Materials & methods* section for details). The magenta, red, and black lines indicate the trial-by-trial variation in the learning effect (mean±s.e.m., N = 12), *x* in the state space model (the quality of the fit (*R*^2^) of the state space model to the data was 0.9394), and the trial-by-trial variation in visuomotor rotation. The thin magenta lines indicate the learning curves for each subject. (b): Estimated retention rate based on 1000 bootstrapped samples of the data obtained in the PoMLab experiments depicted in (a). CI indicates the 95% confidence interval. The horizontal and vertical axes denote the estimated retention rate and the normalized histogram, respectively. (c): Estimated retention rate based on 1000 bootstrapped samples of the data obtained in the PoMLab experiments depicted in (a). (d): Results of the second experiment. The red dotted line indicates the learning curve predicted by the state space model described by parameters that were fit to the data collected during the first experiment (*R*^2^ = 0.8493). (e, f): Estimated retention and learning rate based on 1000 bootstrapped samples of the data obtained in the PoMLab experiments depicted in (d).

The subjects adapted to the abruptly applied perturbation, as shown in Fig 3A. We calculated the learning effect for each set for each subject based on the cursor position at the time of maximal velocity. The deviation between the target angle and the angle of the cursor position *e* was decomposed into two terms, the perturbation *r* and the learning effect *x*, i.e., *e* = *r*−*x*, with *x* calculated as *x* = *r*−*e*. Each data point in Fig 3A and 3B represents the averaged learning effects across four trials, or one set, and all the data for all subjects is shown. The learning effects of the subjects in the CW group were positive while those of the subjects in the CCW group were negative, but we averaged the learning effects by multiplying the learning effects of the CCW group by -1. Subsequently, the set-to-set variation of *x* is referred to as the learning curve.

To evaluate the learning effects that were obtained for each set, we fit a state space model to the obtained learning curve averaged across all participants (the red line in Fig 3A). The state space model can be written as

$$x_{t+1}=Ax_{t}+Be_{t}$$ (1)

with *x*_1_ = 0, where *x*_*t*_ is learning effect at the *t*-th set; *A* is rate of retention (0 ≤ *A* ≤ 1), which describes how memory is retained between sets; and *B* is learning rate (*B* > 0), which describes how fast the movement error can be decreased in each set. In the first PoMLab experiment, *A* and *B* were estimated as 0.881 and 0.356, respectively. Additionally, the correlation coefficient of the fit (*R*^2^) was 0.940. According to this state space fitting, the subjects adapted to the perturbation that was abruptly applied in each set, decreasing the movement error by 36% and forgetting 12% of their current motor memory or, conversely, retaining 88% of their current motor memory. Because the generation of the additional motor command *x* requires effort, an alternative interpretation to the forgetting of motor memory is that the subjects attempted to balance the tradeoff between effort minimization and error minimization [10], i.e., the subjects decreased their movement error by 36% while minimizing their effort, which allowed a 12% increase in movement error in the next set. This procedure of state space model fitting has been successfully used in conventional experiments that employ reaching movements and perturbation learning [9,16]. To estimate 95% confidence intervals (CIs) for parameters *A* and *B*, we collected 1000 bootstrapped samples (Fig 3B, 3C, 3E and 3F). The estimated CIs for *A* and *B* were 0.715 ≤ *A* ≤ 1.000 and 0.175 ≤ *B* ≤ 0.550, respectively.

#### Adaptation to gradually increasing perturbation

If the state space model was a valid way of modeling the learning curves obtained in the PoMLab experiments, we could expect that the values of parameters *A* and *B* estimated in the first experiment could be used to predict learning curves obtained in other experimental settings. Therefore, we conducted another experiment.

In the second experiment, 10 subjects completed 32 practice trials, or 8 practice sets. After the practice trials, the subjects completed 8 baseline trials, followed by 68 trials in which the degree of visuomotor rotation increased in each set (1° increase in each set, with a maximum of 15°). Half of the subjects experienced CW rotation, while the others experienced CCW rotation. To evaluate the subjects’ awareness of the perturbation, the subjects were asked "Please tell me anything you noticed in this experiment" after they completed all of the trials. The subjects adapted to the gradually increasing visuomotor rotation (Fig 3B). The horizontal and vertical axes in Fig 3B denote the set number and the learning effect, respectively. The black line depicts the trial-to-trial variation in visuomotor rotation, and the magenta line depicts the learning curve (mean±s.e.m.). As the perturbation increased in each set, the subjects showed larger learning effects. Furthermore, with *A* = 0.881 and *B* = 0.356, the state space model provided a good prediction of the learning curve (red dotted line, *R*^2^ = 0.849). Notably, these parameters were estimated based only on the learning curves obtained during the first experiment, i.e., the state space model that was fit to the data from the first experiment could predict the learning curves obtained in the second experiment. CIs were estimated as 0.000 ≤ *A* ≤ 1.000 and 0.119 ≤ *B* ≤ 1.946, which included the parameters *A* = 0.881 and *B* = 0.356. Comparison of these CIs to those estimated based on the learning curves for a constant and abruptly applied perturbation suggested that the parameters estimated based on the learning curves for gradually increasing perturbation were more uncertain. When the state space model was fit to the mean learning curve (red line in Fig 3D), with *R*^2^ = 0.909, the retention rate was estimated to be 0.492, and the learning rate was estimated to be 0.801.

Notably, only 1 out of the 10 subjects was aware of the perturbation, suggesting that almost all of the subjects could adapt to the gradually increasing perturbation without being aware of it. In contrast, 5 out of the 12 subjects in the first experiment were aware of the abruptly applied perturbation. Such implicit adaptation in the case of gradually increasing perturbation has been reported in previous studies that employed reaching movements and perturbation learning [17]. Thus, not only the goodness of fit and the prediction power of the state space model could be replicated by the PoMLab experiments; implicit adaptation when the degree of perturbation gradually increased was replicated as well.

### Follow-up experiment

The follow-up experiments were conducted in two sports science classes. Each class had 30 students and was 105 min long. One half of the students (15 subjects) participated in the experiment in one class using 5–8 tablet devices, and the other half participated a couple of weeks later. The subjects completed 32 practice trials, or 8 practice sets. After the practice trials, the subjects completed 8 baseline trials, followed by 68 trials in which the degree of visuomotor rotation increased in each set (1° increase in each set, with a maximum of 15°). They then completed another 8 baseline trials, followed by 44 trials with a constant visuomotor rotation of 15° or -15°. The visuomotor rotation was abruptly applied at the 9th trial, which corresponded to the beginning of the 3rd set, without any notification. Because we focused on whether the students were aware of the gradually increasing perturbation, the subjects first experienced the gradually increasing perturbation and then experienced the abruptly applied perturbation. We used questionnaires to investigate whether the students were aware of the visuomotor rotation. None of the subjects reported being aware of the gradually applied perturbation, and only one student noticed the abruptly applied perturbation.

Thirty students completed the experiments using the Nexus 9 with vibration, 18 students completed the experiments using the Nexus 9 without vibration, and 12 students completed the experiments using the Memo Pad 7 without vibration. First, the data obtained using the Nexus 9 with vibration were compared to the data obtained in the laboratory to discuss whether PoMLab could be used to measure data at any time or place. Second, the data obtained using the Nexus 9 with vibration were compared to the data obtained using the Nexus 9 without vibration to investigate the influence of vibration. Finally, the data obtained using the Nexus 9 without vibration were compared to the data obtained using the Memo Pad 7 without vibration to investigate the influences of the screen size and weight of the tablet.

#### Comparison of the follow-up experiment and the laboratory experiment

**Fig 4:**
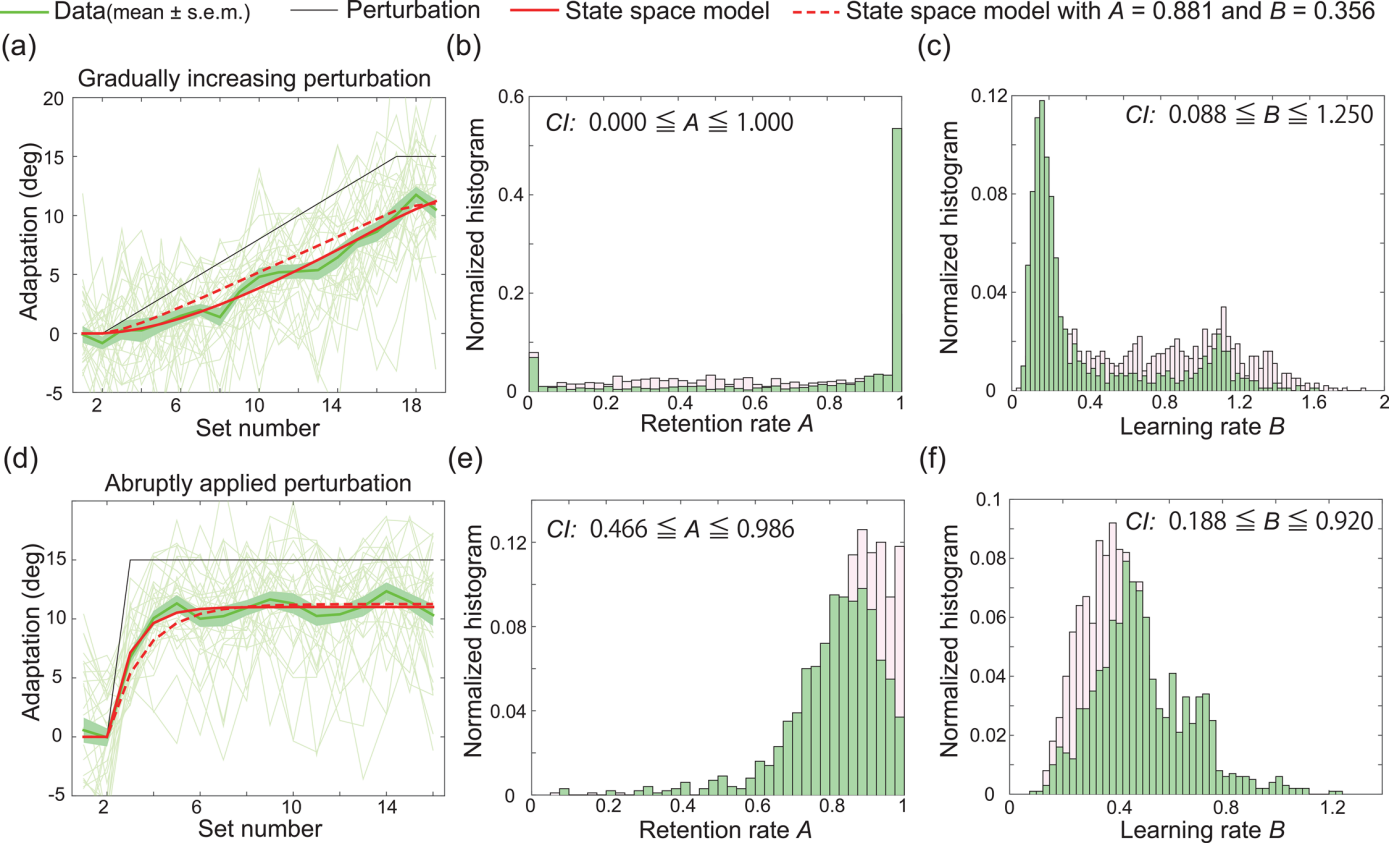
Results of follow-up experiments. (a): Results of the follow-up experiment in which the students experienced gradually increasing perturbation. The horizontal and vertical axes denote the set number and the degree of adaptation, respectively (see *Materials & methods* section for details). The green and black lines indicate the trial-by-trial variation in the learning effect (mean±s.e.m., N = 30) and the trial-by-trial variation in visuomotor rotation, respectively. The red dotted line indicates the learning curve predicted by the state space model described by parameters that were fit to the data collected during the first laboratory experiment (*R*^2^ = 0.9374). The red line indicates the best-fit state space model. The thin green lines indicate the learning curves for each subject. (b, c): Estimated retention and learning rate based on 1000 bootstrapped samples of the data obtained in the PoMLab experiments depicted in (a). The magenta bars indicate the estimated retention and learning rate for the laboratory experiments, as shown in Fig 3B and 3C. (d): Results of the follow-up experiment in which the students experienced a constant perturbation that was applied at a specific trial. (e, f): Estimated retention and learning rate based on 1000 bootstrapped samples of the data obtained in the reaching experiments depicted in (d). The red dotted line indicates the learning curve predicted by the state space model described by parameters that were fit to the data collected during the first laboratory experiment (*R*^2^ = 0.9180).

First, we analyzed the data obtained using the Nexus 9 with vibration in order to compare it with the data obtained in the follow-up experiments at the laboratory. Fifteen subjects experienced CW visuomotor rotation, while the other fifteen subjects experienced CCW visuomotor rotation. Similar to the results of the laboratory experiments, subjects could adapt to both the gradually increasing perturbation and the abruptly applied perturbation (green lines in Fig 4A and 4D). The state space model that was fit to the data obtained during the first laboratory experiment provided a good prediction of the learning curves obtained in the follow-up experiments (red dotted lines in Fig 4A and 4D, *R*^2^ = 0.918 for the learning curve for the gradually increasing perturbation experiment, and *R*^2^ = 0.937 for the learning curve for the abruptly applied perturbation experiment). A CI was determined for each of the parameters fit to the results of the laboratory experiment (Fig 4B, 4C, 4E and 4F). This predictive power suggests that the data obtained during the laboratory and follow-up experiments were similar, validating the utility of the use of PoMLab at any time or place.

When the state space model was fit to the learning curves in Fig 4A and 4D (red lines), *R*^2^ was 0.976 and 0.969, respectively, the retention rates were estimated to be 1.0 and 0.828, and the learning rates were estimated to be 0.148 and 0.476.

#### Influence of vibration

**Fig 5:**
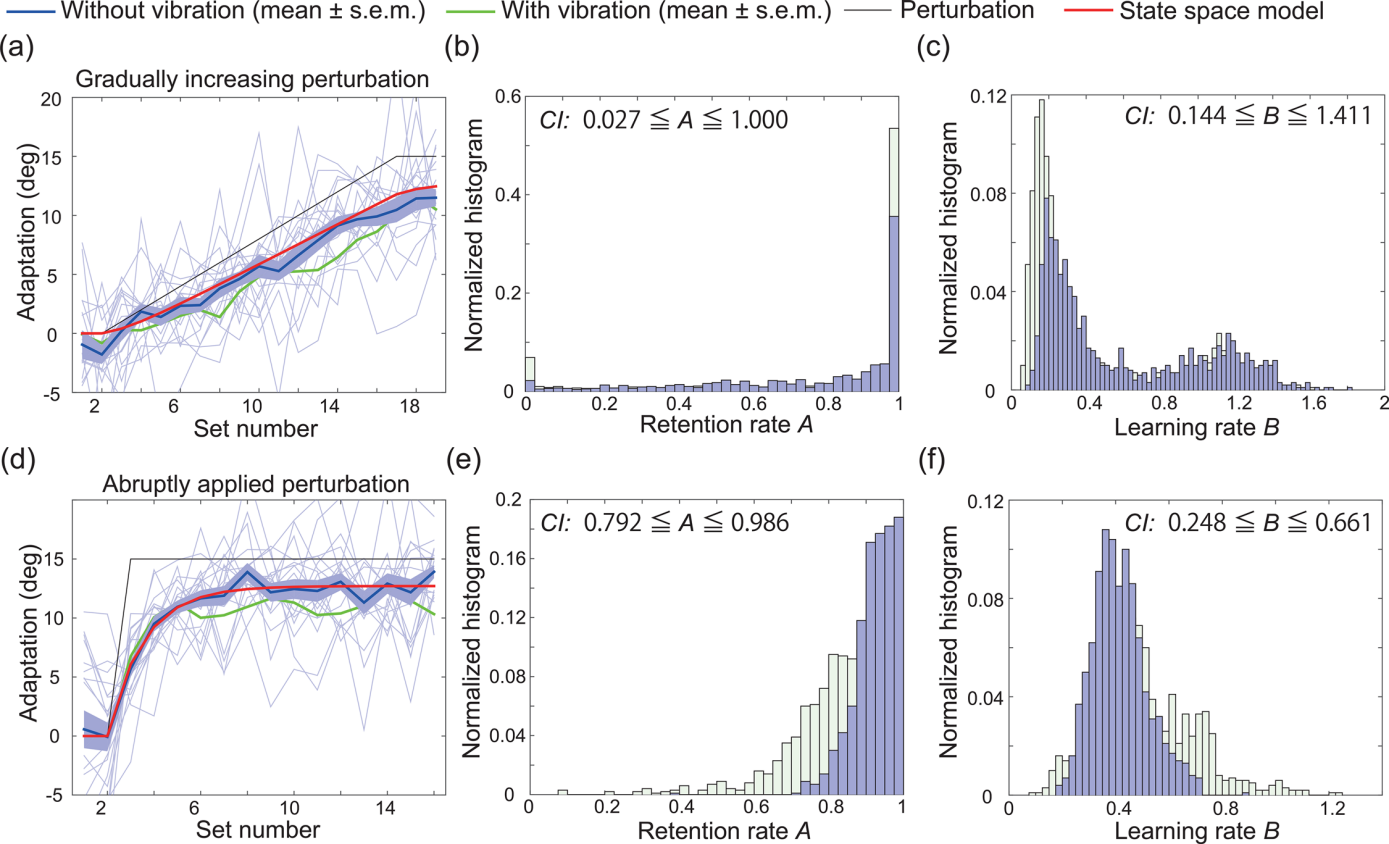
Comparison of the experiments with and without vibration. (a): Results of the follow-up experiment in which the students experienced gradually increasing perturbation with (mean±s.e.m., N = 30, green line) or without (mean±s.e.m., N = 18, blue line) vibration. The horizontal and vertical axes denote the set number and the degree of adaptation, respectively (see *Materials & methods* section for details). The red line indicates the best-fit state space model. The thin blue lines indicate the learning curves for each subject. (b, c): Estimated retention and learning rate based on 1000 bootstrapped samples of the data obtained in the PoMLab experiments depicted in (a). The green bars indicate the estimated retention and learning rate for the follow-up experiments with the Nexus 9 and the vibration function, as shown in Fig 4B and 4C. (d): Results when the students experienced a constant perturbation that was applied at a certain trial. (e, f): Estimated retention and learning rate based on 1000 bootstrapped samples of the data obtained in the reaching experiments depicted in (d).

In the laboratory and follow-up experiments described above, the subjects experienced vibrations when the cursor contacted the targets. Because a vibrating function is not available in several smart devices, we investigated the influence of vibration on the learning curves. Eighteen students completed the same PoMLab task that was used in the follow-up experiments described above without experiencing any vibration. Fig 5A and 5D show the learning curves obtained without any vibration (blue lines) and those obtained with vibration (green lines). Fig 5B, 5C, 5D and 5E depict the bootstrapped retention rate, the bootstrapped learning rate, and the corresponding CIs. When the state space model was fit to the learning curves in Fig 5A and 5D (red lines), *R*^2^ was 0.978 and 0.976, respectively, the retention rates were estimated to be 0.910 and 0.927, and the learning rates were estimated to be 0.344 and 0.405.

#### Comparison of the different tablet devices

**Fig 6:**
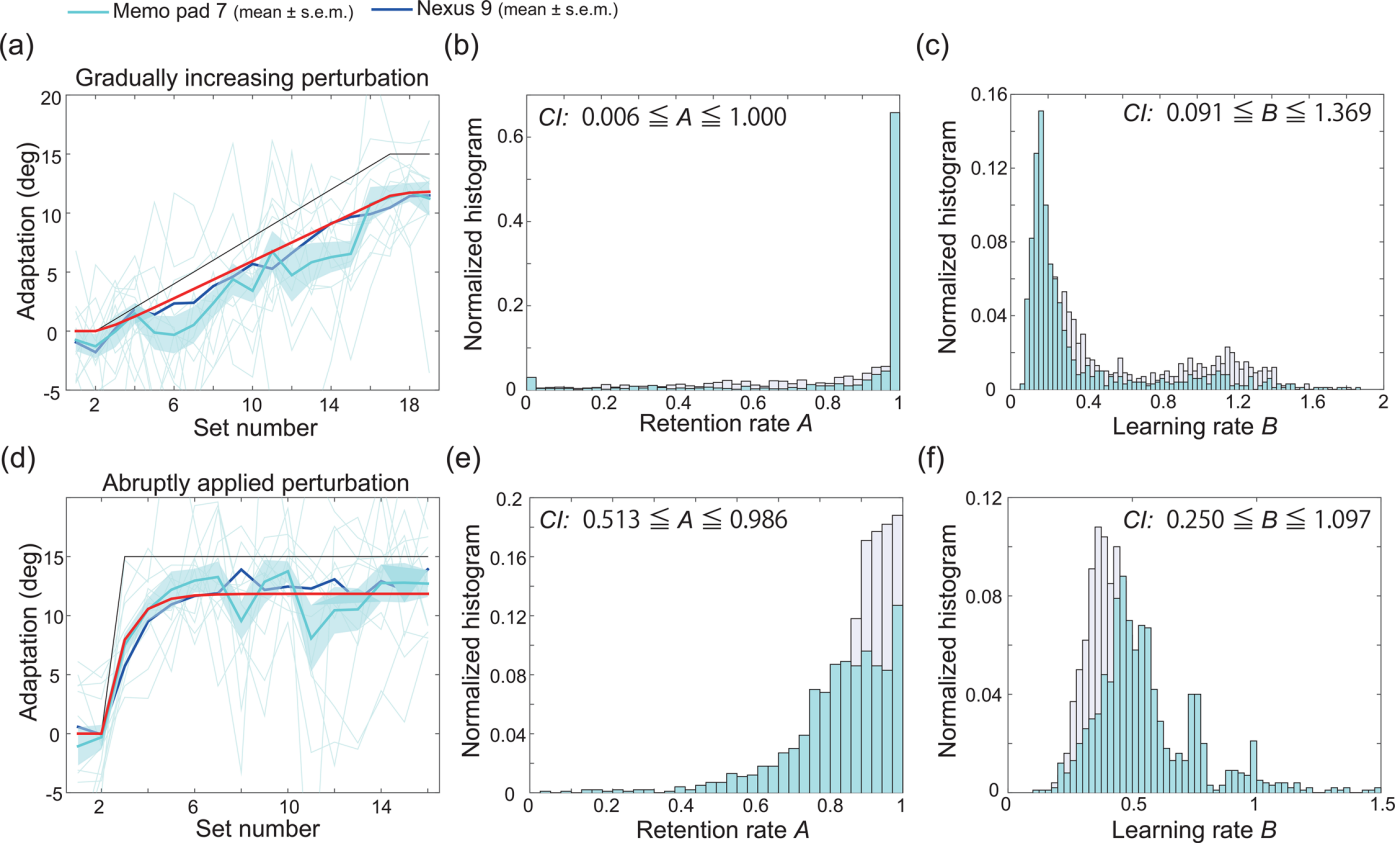
Comparison of the results obtained using different tablet devices. (a): Results of the follow-up experiment in which the students experienced gradually increasing perturbation using a Nexus 9 (mean±s.e.m., N = 18, blue line) or Memo Pad 7 (mean±s.e.m., N = 12, cyan line). None of the students experienced vibration. The horizontal and vertical axes denote the set number and the degree of adaptation, respectively (see *Materials & methods* section for details). The red line indicates the best-fit state space model. The thin cyan lines indicate the learning curves for each subject. (b, c): Estimated retention and learning rate based on 1000 bootstrapped samples of the data obtained in the PoMLab experiments depicted in (a). The blue bars indicate the estimated retention and learning rate for follow-up experiments with the Nexus 9 and without the vibration function, as shown in Fig 5B and 5C. (d): Results when the students experienced a constant perturbation that was applied at a certain trial. (e, f): Estimated retention and learning rate based on 1000 bootstrapped samples of the data obtained in the reaching experiments depicted in (d).

Because Unity3D enabled us to build the PoMLab application for use with various types of tablets, we investigated the influence of the weight and screen size of the tablet device. Twelve students used a Memo Pad 7, which does not have a vibration function, to complete the same PoMLab task used in the follow-up experiments described above. Fig 6A and 6D show the learning curves obtained using the Nexus 9 without any vibration (blue lines) and those using the Memo Pad 7 (cyan lines). Fig 6B, 6C, 6D and 6E depict the bootstrapped retention rate, bootstrapped learning rate, and corresponding CIs. When the state space model was fit to the learning curves in Fig 6A and 6D (red lines), *R*^2^ was 0.929 and 0.883, respectively, the retention rates were estimated to be 1.000 and 0.859, and the learning rates were estimated to be 0.159 and 0.530.

### Comparison of PoMLab experiment and reaching experiment

Finally, we compared the learning curves obtained in the PoMLab experiments to those obtained in the experiments with reaching movements, a golden standard for motor learning experiments. Fourteen subjects experienced both PoMLab and reaching experiments with gradually increasing perturbation. Half of the participants experienced the PoMLab experiments first, while the other half experienced the reaching experiment first. We gradually increased the perturbation to eliminate the influence of the subjects’ awareness of the perturbation on learning. The subjects completed 64 practice trials, or 16 practice sets. After the practice trials, the subjects completed 8 baseline trials, followed by 84 trials in which the degree of visuomotor rotation increased in each set (1° increase in each set, with a maximum of 15°). Half of the subjects experienced CW rotation, while the others experienced CCW rotation.

**Fig 7:**
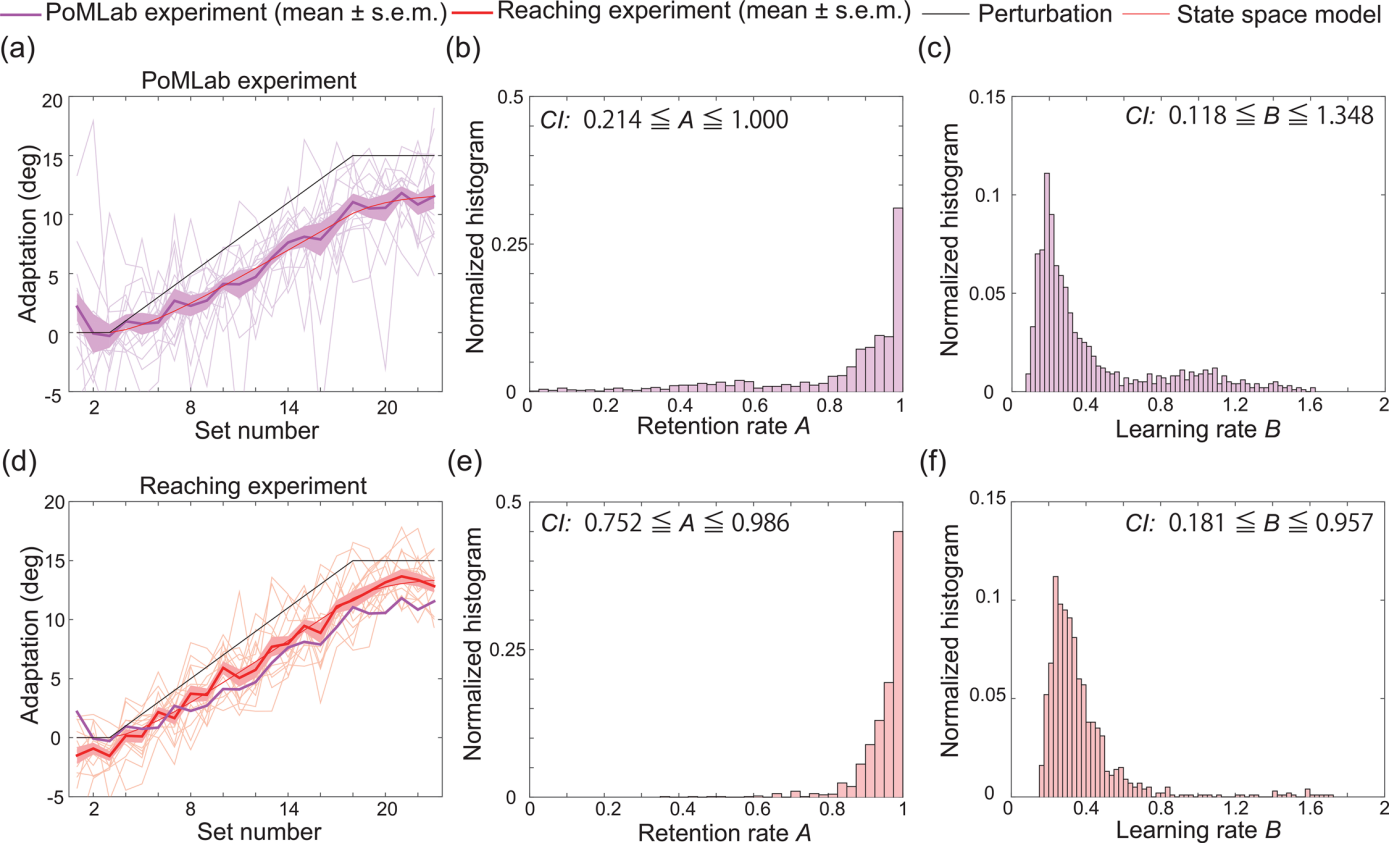
Comparison of the PoMLab experiments and the reaching experiments. (a): Results of the PoMLab experiment in which the subjects experienced gradually increasing perturbation using a Nexus 9 without vibration (mean±s.e.m., N = 14, magenta line). The horizontal and vertical axes denote the set number and the degree of adaptation, respectively (see *Materials & methods* section for details). The red line indicates the best-fit state space model. The thin magenta lines indicate the learning curves for each subject. (b, c): Estimated retention and learning rate based on 1000 bootstrapped samples of the data obtained in the PoMLab experiments depicted in (a). (d): Results of the reaching experiment in which the subjects experienced gradually increasing perturbation (mean±s.e.m., N = 14, red line). (e, f): Estimated retention and learning rate based on 1000 bootstrapped samples of the data obtained in the reaching experiments depicted in (d).

Fig 7A and 7D depict the learning curves obtained in the PoMLab and reaching experiments, respectively. Fig 7B, 7C, 7E and 7F depict the bootstrapped retention rate, the bootstrapped learning rate, and the corresponding CIs. Although the estimated learning rate tended to be similar for the two kinds of experiments (Fig 7C and 7F), the estimated retention rate tended to be lower for the PoMLab experiments than for the reaching experiments (Fig 7B and 7E).

When the state space model was fit to the learning curves in Fig 7A and 7D (thin red lines), *R*^2^ was 0.974 and 0.980, respectively, the retention rates were estimated to be 0.932 and 0.967, and the learning rates were estimated to be 0.250 and 0.309.

Notably, 3 of the 14 subjects were aware of the perturbation in the reaching experiment, but no subject was aware of the perturbation in the PoMLab experiment. This result suggests that the perturbation in the PoMLab experiment was less noticeable than that in the reaching movement experiment.

### Additional statistical assessment

Because the state space model is a parametric model, we reanalyzed all of the experimental data based on a nonparametric method that was used in a previous study [18]. Initial learning was defined as the averaged learning effects across the initial 3 sets after the perturbation was imposed. The asymptote was defined as the averaged learning effects across the last 3 sets in each experiment.

**Fig 8:**
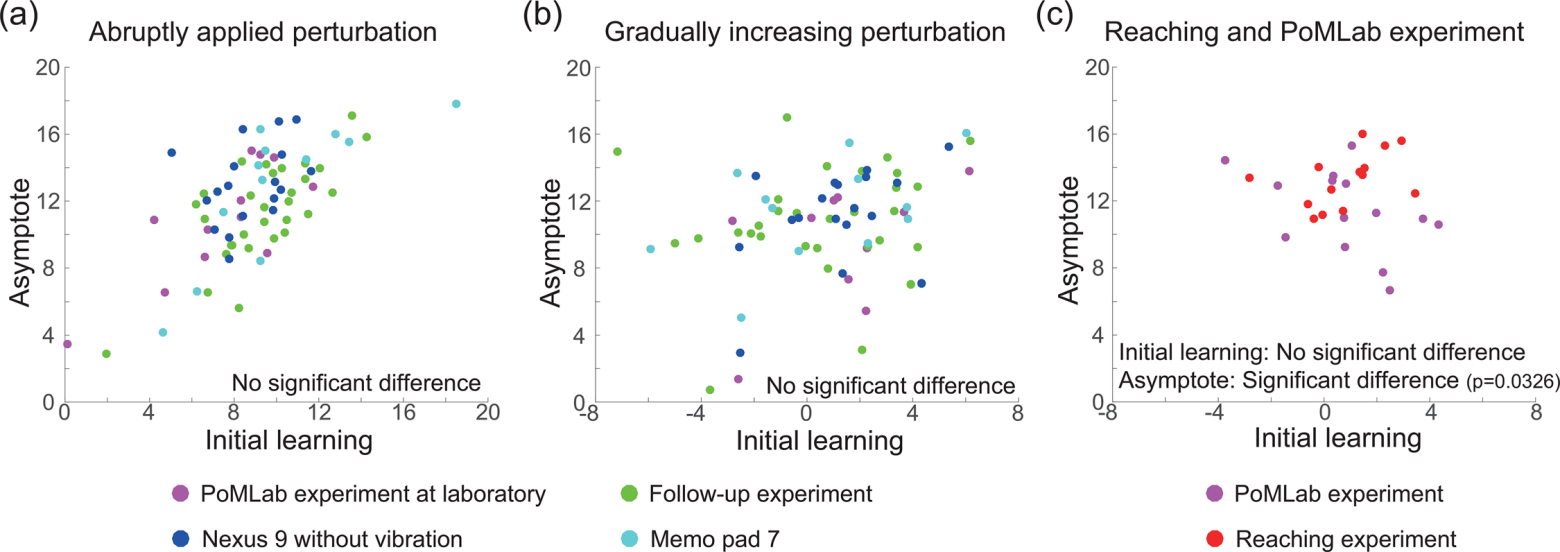
Assessment of experimental data based on a nonparametric method. (a): Initial learning and asymptote of each participant when perturbation was abruptly applied. Magenta, green, blue, and cyan dots indicate the values for the PoMLab experiment at the laboratory, the follow-up experiment, the PoMLab experiment with Nexus 9 without vibration, and the PoMLab experiment with Memo Pad 7. (b): Initial learning and asymptote of each participant when perturbation was gradually increased. (c): Initial learning and asymptote of each participant with perturbation in the PoMLab and reaching experiments.

Fig 8(A) and 8(B) denote the initial learning and asymptote of each participant when perturbation was abruptly applied and those when perturbation was gradually increased, respectively. Magenta, green, blue, and cyan dots indicate the values of the PoMLab experiment in the laboratory, the follow-up experiment, the PoMLab experiment with Nexus 9 without vibration, and the PoMLab experiment with Memo Pad 7. No significant difference was observed in these values (p > 0.05, Wilcoxon signed-rank test).

Fig 8(C) denotes the initial learning and asymptote of each participant in the PoMLab and reaching experiments. A significant difference was found in the asymptote (p = 0.0326), no significant difference was found in initial learning (p > 0.05).

## Discussion

We developed a novel application, the POrtable Motor learning LABoratory (PoMLab), that can be used to conduct motor learning experiments using smart devices, enabling the experiments to be conducted at any time or place, for free. These advantages can eliminate the requirements of conventional motor learning experiments for large and expensive experimental devices, i.e., space and monetary budget, as well as the burden for subjects to travel to the laboratory. This burden is severe when the subjects involve patients or when a large amount of data is necessary. For example, for evaluations of individual differences, our PoMLab application can eliminate the burden because it enables motor learning experiments to be conducted at any time and place. We validated the use of our PoMLab application as a tool to conduct motor learning experiments by demonstrating that PoMLab experiments could reproduce the results obtained in conventional experiments involving reaching movements and perturbation learning and in which subjects were required to decrease sensory prediction errors to successfully complete the imposed task. First, we demonstrated that the subjects showed trial-by-trial adaptation to a constant visuomotor rotation that was abruptly applied at a certain trial (Fig 3A). Second, the obtained learning curves were well fit by a state space model, a type of model that is widely used to describe the motor learning process (red line in Fig 3A). Third, the state space model that was fit to the data obtained during the first experiment could predict the learning curve obtained in the second experiment (red dotted line in Fig 3D). This prediction power shows that the state space model could be used to model and predict learning curves in the PoMLab experiments. Fourth, the subjects adapted to the gradually increasing perturbation without being aware of the perturbation. These experimental results have been reported for conventional experiments that employed reaching movements and perturbation learning [9,17], suggesting that PoMLab could be an alternative to conventional experiments. Fifth, the learning curves obtained in the classrooms, which were not perfectly isolated and insulated, could be predicted well by the state space model that was described by parameters that were fit to the data obtained during the first laboratory experiment, further demonstrating that PoMLab can be used to conduct motor learning experiments at any time or place. Finally, to further validate the PoMLab experiments, we had the same set of subjects complete both PoMLab and reaching experiments (Fig 7). This direct comparison revealed that the estimated retention rate *A* tended to be lower, and the confidence interval was wider (i.e., the estimation reliability was lower) for the PoMLab experiments than for the reaching experiments (Fig 7B and 7E), but adaptation without any awareness was observed more frequently in the PoMLab experiments than in the reaching experiments. Furthermore, our PoMLab application can be freely shared with many people, including researchers in the field of motor control and learning, doctors, and physical therapists. It is also possible for others to further develop and improve PoMLab because Unity3D can be used to code programs on both Mac and Windows computers, and the applications can be built for Android, iOS, Mac, Windows, and many other types of operating systems. It is important to understand the pros and cons of conventional laboratory experiments using manipulandum and field experiments using PoMLab. Because manipulandum is a very sophisticated experimental system, if precise and reliable measurements are needed, manipulandums should be used. Instead, if a high volume of data from many subjects and patients who cannot travel to the laboratory is required, PoMLab can be a convenient tool as long as the limitations of the method are recognized.

**Fig 9:**
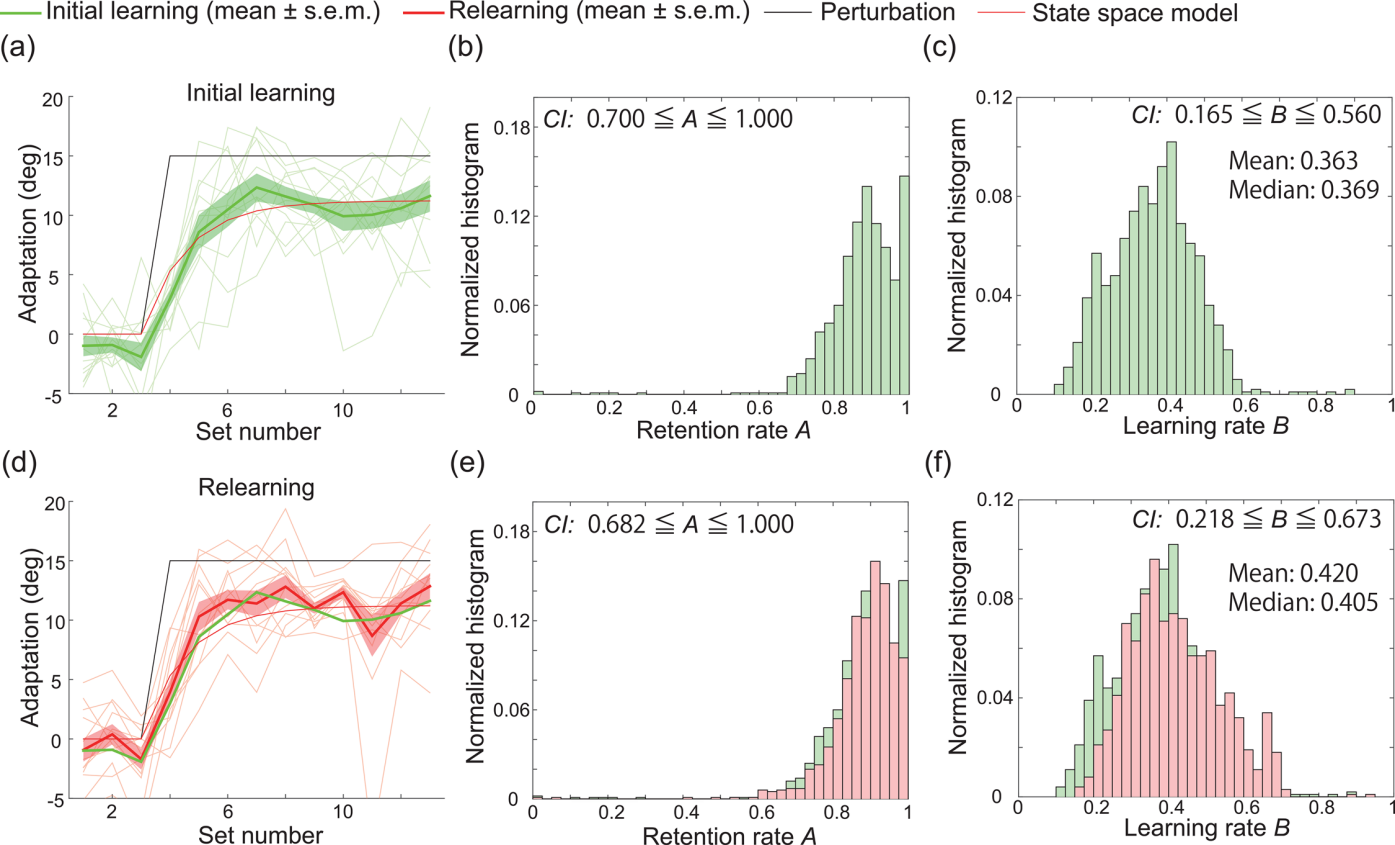
Comparison of the results for the initial learning and relearning phases. (a): Results of a pilot experiment to investigate savings; in this experiment, the subjects experienced abruptly applied perturbation using a Nexus 9 with vibration. The green line shows the learning curve for the initial learning phase (mean±s.e.m., N = 12). Fig 8A–8C are the same as Fig 3A–3C. The difference in the confidence intervals between Figs 3B and 8B or 3C and 8C is due to the randomness of the bootstrapped resampling. (d): The learning curve for the relearning phase (mean±s.e.m., N = 12). In this pilot study, after a short break, 12 subjects experienced the same perturbation as in the initial learning phase. The green line depicts the learning curve for the initial learning phase. (e, f): The red bars indicate the estimated retention and learning rates for the relearning phase, and the green bars indicate those for the initial learning phase.

One limitation of PoMLab experiments is the size of the workspace. The small workspace limits the size of the cursor and target, resulting in some residual error. In conventional experiments with reaching movements, a large workspace is used, e.g., reaching movements of 10 cm are frequently employed [7,9]. In such workspaces, the subjects need to generate a nearly straight path to reach for targets, which means that the size of the cursor and target do not significantly affect the learning curves. In contrast, the workspace in PoMLab is smaller than that in conventional experiments. On the Nexus 9, the distance between the center circle and the target was 3.5 cm; as a result, a deviation of 7.38° from a straight line is allowed when subjects attempt to move the cursor to the target (please refer to Fig 2C). In fact, there was approximately 5° of residual error in the learning curves (Figs 3A, 3B, 4A and 4B). In the framework of a state space model, residual error causes a small retention rate [19], meaning that the retention rate in the PoMLab experiments was likely an underestimate. This residual error, or small retention rate, was likely the effect of such a small workspace. How to address the small workspace and decrease residual error is thus a problem that remains to be resolved in the PoMLab application. However, the state space model that was fit to the learning curves obtained in the first laboratory experiment could predict not only the learning curves obtained during the second laboratory experiment (red dotted line in Fig 3D) but also the learning curves obtained in the follow-up experiments (red dotted lines in Fig 4A and 4D). Despite the notable residual error, these results indicate that the state space model can be used to quantitatively compare learning curves obtained in PoMLab experiments. Further, we could confirm a slight savings from our pilot studies (Fig 9), consistent with previous observations that learning is faster during the relearning phase than during the initial learning phase when subjects adapt to the same perturbation twice [7,16]. When the state space model was fit to the learning curves obtained in the initial learning phase (Fig 9A) and in the relearning phase (Fig 9D), *R*^2^ was 0.944 and 0.931, respectively (thin red lines), the retention rates were estimated to be 0.880 and 0.887, and the learning rates were estimated to be 0.355 and 0.411. There are several possible explanations for why only a slight savings was observed in the PoMLab pilot experiments. For example, it may not be possible to observe savings for small amounts of visuomotor rotation [20] (15° rotation in our PoMLab experiments), or considerable savings may be observed only for familiar movements like reaching movements and not for tilting movements. We need to further investigate differences between conventional paradigms of motor learning and PoMLab.

We utilized the advantages of PoMLab, including its portability, convenience, and the ability to conduct experiments at any time or place, to conduct follow-up experiments. An important finding of these experiments was that the state space model that was defined by parameters that were estimated based on the data obtained during the laboratory experiments could provide a good prediction of the learning curves obtained in the follow-up experiments. This result indicated that the data obtained in the follow-up experiments could be fairly compared to that obtained in the laboratory experiments, showing that PoMLab could be used at any time or place. Due to this convenience, PoMLab may be a useful educational tool for cognitive science, neuroscience, or programming classes. In fact, the students reported that completing the task stimulated their interest and led to greater understanding than simply reading a textbook. Students can participate in motor learning experiments, learn programming skills to analyze the obtained data, and learn how to fit mathematical models (i.e., state space models) to the obtained data. This type of active learning about motor learning can be difficult to achieve through the use of conventional laboratory experiments involving manipulandums because considerable time is required for all of the students in a class to experience the motor learning experiment. In contrast, PoMLab allows all of the students in a class to experience a motor learning experiment at once by using their smartphones or tablets. PoMLab can thus be not only a useful experimental tool for patients and the elderly but also a powerful educational tool in classes.

Figs 4 and 5 showed that vibration did influence the learning curves to some extent. Abruptly applying the perturbation caused notable differences in the learning curves, including the lower bound of the CI for the estimated retention rate and the upper bound of the CI for the learning rate, indicating that the estimated retention rate tended to be larger and the estimated learning rate tended to be smaller in the PoMLab experiments without vibration compared to those with vibration. Because, as mentioned above, the cursor can contact the target even if there is approximately 7° of residual error, the subjects experienced the vibration beginning with the 4^th^ set in Fig 5D. One interpretation of the influence of the vibration is that the subjects chose to repeat the actions employed in the 4^th^ set because they received strong reinforcement that they could achieve the task through the vibration, resulting in a larger residual error and smaller retention rate than the PoMLab experiments without vibration. In contrast, without any vibration, the subjects could confirm that they achieved the task only through the target’s disappearance. As a result, they explored more straight cursor movements, resulting in a smaller residual error and larger retention rate. Because the subjects in the PoMLab experiments without vibration could explore more straight cursor movements without requiring a large number of trials, the smaller learning rate could also originate from such exploration.

Differences between the learning curves obtained with the Nexus 9 and Memo Pad 7 are evident by comparing Figs 5 and 6. One example is the lower limit of the CI of the retention rate estimated from the learning curves when the perturbation was abruptly applied; the estimated retention rate was lower for the PoMLab experiments conducted with the Memo Pad 7 compared to those conducted with the Nexus 9. Another difference is the fluctuation in the learning curves obtained with the Memo Pad 7. We can infer that the fluctuation is due to the light weight of the Memo Pad 7. When we hold tablet devices, it is difficult to hold the devices completely statically, likely because of motor command- or muscle activity-related noise, and some fluctuations in the location of the cursor can be observed in PoMLab experiments. This fluctuation is considerable when the tablet device is light. The low-pass filter was thus introduced as part of the settings of PoMLab (see *Materials & Methods* section), with α = 0.98. The larger the value of the α parameter, the smaller the fluctuation. The decision to set α = 0.98 was based on what was an appropriate value for the experiments with the Nexus 9, which is a heavier device than the Memo Pad 7. It can thus be speculated that a using a larger α value for experiments conducted with the Memo Pad 7 can eliminate the fluctuation in the learning curves. How to determine an appropriate α for each tablet device is a problem to be solved in a future project.

Because a powerful application of PoMLab is the ability to conduct motor learning experiments using patient populations, we need to investigate how to fix the following problems. The first problem is the weight of smart devices. The weight of the Nexus 9 is 425 grams, and it is not difficult for healthy subjects to tilt the tablet many times. Nevertheless, whether the Nexus 9 tablet is appropriate for patients is not self-evident. In fact, some of the subjects in our PoMLab experiments reported a small degree of arm muscle fatigue. Although our PoMLab experiments can also be conducted using tablet devices that are lighter than the Nexus 9, such as the Memo Pad 7 (295 g), the learning curves obtained using the lighter tablet devices showed more oscillatory behavior (Fig 6). We need to further investigate the type of tablet and the value of the low-pass filter parameter α that will be the best for the conduction of PoMLab experiments with patients. We also need to determine whether support devices are necessary and the number of trials that is suitable for patients. The second problem is the duration of target presentation. In our PoMLab experiments, the subjects were required to move the cursor to contact the target within 1.5 seconds. Some of the subjects in our PoMLab experiments reported feeling that it was difficult to move the cursor within this time frame at the beginning of the experiment. After practicing, all of the subjects could successfully perform the reaching task without any difficulty. However, a duration that is appropriate for patients remains to be determined. We doubt that the duration used here is too short to prevent all patients from successfully performing the reaching tasks; however, it is necessary to determine an appropriate duration for each patient. Notably, conventional studies have investigated motor learning using a feedforward controller or appropriately fast reaching movements. We thus did not focus on presenting the targets for long periods of time. After resolving these outstanding issues, our PoMLab is likely to be a powerful tool to investigate the neural mechanisms of motor learning and effective rehabilitation methods. Because some computational models have proposed effective rehabilitation methods [21,22], it is desirable to investigate these proposals by conducting motor learning experiments in a large number of patients using our PoMLab application.

Further improvements and applications of PoMLab are possible. One possibility is the use of both an accelerometer and gyroscope, although only the accelerometer was used in the current version of PoMLab. Kalman or complementary filters could also be employed to increase the reliability of the PoMLab application. The collection of data from thousands of participants using Amazon mechanical turk (https://aws.amazon.com/jp/mturk/) or similar systems is another possibility. The generalization of motor learning effects from one trained movement to other untrained movements was investigated in a previous study through the use of smartphones and laboratory setting [23]. The concept of this previous study was similar to that of our study, but our PoMLab application has some advantages over the previous method: the motor learning experiments can be conducted anywhere at any time, and the PoMLab application is multiplatform. However, the generalization of PoMLab-based learning should be investigated. Rehabilitation is another possible application for PoMLab. Recent studies have attempted to perform rehabilitation at home, especially through the use of tablet devices, tablet games, or other devices that can connect to the tablet through Bluetooth [24,25]. We need to investigate whether our PoMLab application is effective for home-based rehabilitation.
